# Exosomal microRNA-92b Is a Diagnostic Biomarker in Breast Cancer and Targets Survival-Related MTSS1L to Promote Tumorigenesis

**DOI:** 10.3390/ijms25021295

**Published:** 2024-01-20

**Authors:** Jung-Yu Kan, Shen-Liang Shih, Sheau-Fang Yang, Pei-Yi Chu, Fang-Ming Chen, Chung-Liang Li, Yi-Chia Wu, Yao-Tsung Yeh, Ming-Feng Hou, Chih-Po Chiang

**Affiliations:** 1 Division of Breast Oncology and Surgery, Department of Surgery, Kaohsiung Medical University Hospital, Kaohsiung Medical University, Kaohsiung 80756, Taiwan; kan890043@gmail.com (J.-Y.K.); slshih1@gmail.com (S.-L.S.); fchen@kmu.edu.tw (F.-M.C.); kmuhduty@gmail.com (C.-L.L.); yichiawu@kmu.edu.tw (Y.-C.W.); 2 Department of Pathology, Kaohsiung Medical University Hospital, Kaohsiung Medical University, Kaohsiung 80756, Taiwan; 3 Department of Pathology, Show Chwan Memorial Hospital, Changhua 50544, Taiwan; chu.peiyi@msa.hinet.net; 4 Division of Plastic Surgery, Department of Surgery, Kaohsiung Medical University Hospital, Kaohsiung Medical University, Kaohsiung 80756, Taiwan; 5 Department of Medical Laboratory Sciences and Biotechnology, Fooyin University, Kaohsiung 83130, Taiwan; glycosamine@yahoo.com.tw; 6 Department of Biomedical Science and Environmental Biology, College of Life Science, Kaohsiung Medical University, Kaohsiung 80756, Taiwan

**Keywords:** exosomes, breast cancer, miRNAs, miR-92b, MTSS1L

## Abstract

Exosomal microRNAs (miRNAs) are novel, non-invasive biomarkers for facilitating communication and diagnosing cancer. However, only a few studies have investigated their function and role in the clinical diagnosis of breast cancer. To address this gap, we established a stable cell line, MDA-MB-231-CD63-RFP, and recruited 112 female participants for serum collection. We screened 88 exosomal miRNAs identified through microarray analysis of 231-CD63 and literature screening using real-time PCR; only exosomal miR-92b-5p was significantly increased in patients with breast cancer. It had a significant correlation with stage and discriminated patients from the control with an AUC of 0.787. Exosomal miR-92b-5p impacted the migration, adhesion, and spreading ability of normal human mammary epithelial recipient cells through the downregulation of the actin dynamics regulator MTSS1L. In clinical breast cancer tissue, the expression of MTSS1L was significantly inversely correlated with tissue miR-92b-5p, and high expression of MTSS1L was associated with better 10-year overall survival rates in patients undergoing hormone therapy. In summary, our studies demonstrated that exosomal miR-92b-5p might function as a non-invasive body fluid biomarker for breast cancer detection and provide a novel therapeutic strategy in the axis of miR-92b-5p to MTSS1L for controlling metastasis and improving patient survival.

## 1. Introduction

Breast cancer is the most common cancer and the leading cause of cancer-related deaths in women worldwide. The overall 5-year survival rate with early-stage (stage 0-II) breast cancer is about 90%, whereas for later-stage (stage III–IV) breast cancer, it is about 20–60% [1]. Despite improvements in diagnosis and targeted therapy, breast cancer is still the most prevalent cancer in women and lacks an effective early detection marker. The urgency to identify circulating, real-time, and non-invasive biomarkers for breast cancer detection cannot be overemphasized.

MicroRNAs are non-coding single-strand 21–24 nucleotide RNAs that bind to the 3′-untranslated region (3′-UTR) of the target gene and cause negative regulation by either translation repression or mRNA cleavage [2]. It has been estimated that more than 60% of human genes are regulated by miRNAs [3], and these properties render miRNAs vital in many biological processes and cancer development [4,5,6]. Moreover, circulating miRNAs exist in body fluids as the major non-invasive biomarkers for cancer detection [7,8]. These cell-free miRNAs in serum or plasma were initially reported to be stable in circulation [9]. Later studies demonstrated that the majority of the cell-free miRNAs are packaged into exosomes [10,11] that protect them from degradation. Therefore, exosomal miRNAs are the major circulating miRNAs of non-invasive biomarkers.

Exosomes are small secreted membrane vesicles with a diameter of 30–100 nm. The biogenesis of exosomes is derived from the inward budding of the cellular membrane to form an early endosome and further sorted to late endosomes of intraluminal vesicles (ILVs). These form a multivesicular body (MVB) and are released when they fuse with the cell membrane [12,13]. In 2007, a crucial study brought the field of exosomes into focus. The author found that exosomes play crucial roles in cellular communication to deliver genetic information, including mRNA and miRNAs. The study also provided the first evidence of miRNAs in exosomes [14]. Exosomal miRNAs are stable and enriched in cancer patients, making them ideal, novel, non-invasive biomarkers for diagnosis and a communication bridge in breast cancer [15]. However, few studies have demonstrated the role of exosomal miRNAs in the diagnosis of breast cancer, and their functions have not yet been fully understood. Therefore, this study aimed to establish non-invasive body fluid biomarkers from exosomal miRNAs for breast cancer detection and to elucidate the function of exosomal miRNAs in vitro and in clinical tissues.

## 2. Results

### 2.1. Characterization of Isolated Exosomes and Expression Profile of Exosomal miRNA

**Figure 1:**
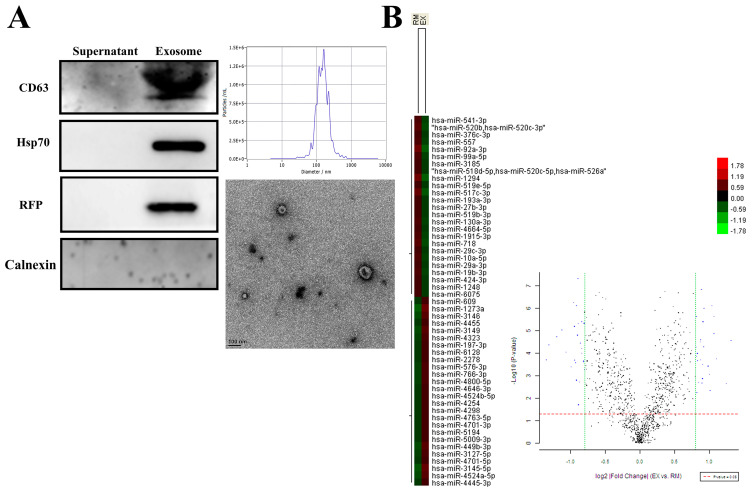
Characterization of the exosomes isolated from 231-CD63 and miRNA profiles after uptake by RMF-EG. (**A**) Isolation of exosomes from a culture medium is confirmed by Western blots, showing strong expression of exosomal markers for CD63, Hsp70, and RFP and absence of negative exosomal marker for Calnexin. NTA analysis and TEM also demonstrate that the isolated portion conforms to the size distribution, concentrations, and morphology of exosomes. (**B**) Total RNA is extracted using TRIzol reagent after 48 h of exosome uptake and analyzed using miRNA arrays. Twenty-six miRNAs reveal increased expression (indicated by red colors in the EX column and blue dots in the right quadrant of Volcano plot) after the uptake of exosomes from 231-CD63 (EX).

To investigate the potential exosomal miRNA from breast cancer, we first used a cell line model and selected the highly metastatic breast cancer cell line MDA-MB-231 to establish a stable cell line, MDA-MB-231-CD63-RFP (231-CD63), which expresses the exosomal marker CD63 tagged with red fluorescent protein (RFP) to track exosomes. As shown in Figure 1A, the secretion of exosomes from 231-CD63 was confirmed using Western blots to demonstrate that the extraction part expressed specific exosomal markers for CD63, Hsp70, and RFP but did not detect the negative exosomal marker for Calnexin. In addition, nanoparticle tracking analysis (NTA) demonstrated the size distribution and concentrations of exosomes. The morphology of exosomes was confirmed using transmission electron microscopy (TEM), indicating that the isolation part was an exosome and was not contaminated with other cellular components.

The major function of exosomes is to deliver messengers within exosomes, such as miRNA. To study the exosomal miRNA from breast cancer, exosomes isolated from 231-CD63 were delivered into the recipient cells, RMF-EG After 48 h, the recipient cells were extracted using TRIzol reagents and analyzed using miRNA arrays. As shown in Figure 1B, compared to the control of RMF-EG (RM), 26 miRNAs revealed increased expression (red colors in the column of EX and blue dots in the right quadrant of the Volcano plot) after the uptake of exosomes from 231-CD63 (EX), and these miRNAs were potential candidates for further literature search and human serum analysis.

### 2.2. Exosomal miR-92b-5p Was Significantly Increased in a Different Stage of Breast Cancer Patients

**Figure 2:**
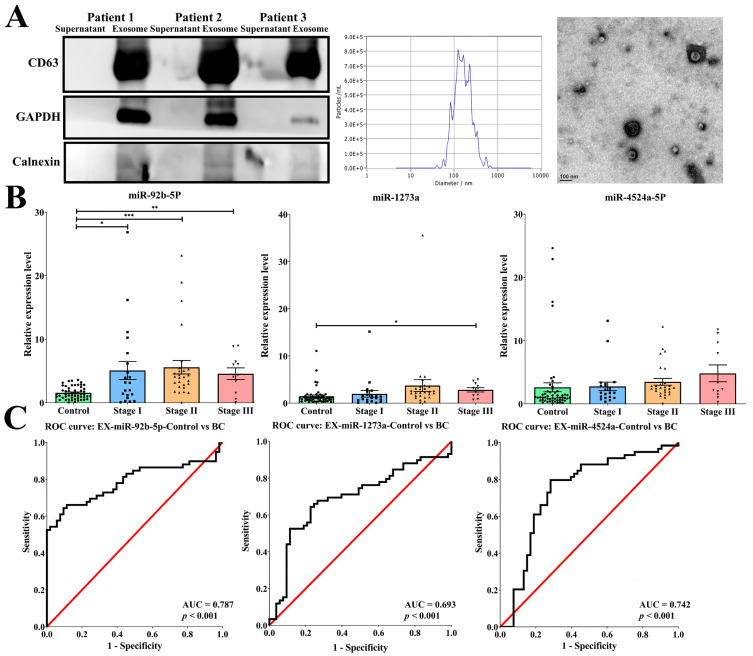
Exosomal miR-92b-5p functions as a non-invasive body fluid biomarker for breast cancer detection. (**A**) Isolation of exosomes from the serum of breast cancer patients is confirmed by Western blots, showing strong expression of exosomal markers for CD63 and GAPDH and the absence of negative exosomal marker for Calnexin. NTA analysis and TEM also demonstrate that the isolated portion conforms to the size distribution, concentrations, and morphology of exosomes. (**B**) Among the three candidate exosomal miRNAs, miR-92b-5p exhibits significant increase in different stages (stage I: N = 21, 5.08-fold increase; stage II: N = 27, 5.58-fold increase; stage III: N = 11, 4.57-fold increase) in patients with breast cancer compared with controls (N = 53). The *p*-value is estimated using a two-tailed *t*-test, and a *p*-value of less than 0.05 (denoted as * *p* < 0.05, ** *p* < 0.01, and *** *p* < 0.001) denotes statistical significance. (**C**) The receiver operating characteristic (ROC) curve shows that miR-92b-5p reached an acceptable level of discrimination, with an area under the curve (AUC) of 0.787 for distinguishing patients with breast cancer from controls.

The main goal of this study was to identify a specific exosomal miRNA in the body fluid that functions as a biomarker for distinguishing patients with breast cancer from controls and elucidate the function alternation of the target gene by exosomal miRNA. Initially, we randomly isolated exosomes from the serum of three patients, and the isolation part strongly expressed exosomal markers for CD63 and GAPDH but did not detect the negative exosomal marker for Calnexin. NTA analysis and TEM demonstrated the size distribution, concentrations, and morphology of the exosomes (Figure 2A).

Next, we screened a total of 88 exosomal miRNAs in the partial breast cancer patient’s serum using real-time PCR. These candidate miRNAs were selected from microarray data and the literature crucial for their significance in breast cancer, and were upregulated in the microarray. As shown in Table 1, a Ct value of ≥35 was considered a low expression level in patients with breast cancer. Finally, a total of nine miRNAs with a Ct value < 35 were selected as candidate serum exosomal miRNAs. In the next experiment, we found that the levels of serum exosomal miRNAs miR-92b-5p, miR-1273a, and miR-4524a-5p were significantly increased with the progression of the stage of breast cancer. With the accumulation of samples, only exosomal miR-92b-5p met the goal of significant differential expression versus control donors (stage I: 5.08-fold increase; stage II: 5.58-fold increase; stage III: 4.57-fold increase; Figure 2B). Exosomal miR-92b-5p also exhibits a significant increase in the ER and HER-2 subtypes in patients with breast cancer compared with controls (Supplementary Figure S1). These exosomal miRNAs were further confirmed using the ROC curve, and only exosomal miR-92b-5p reached an acceptable discrimination of the AUC, which was 0.787 (*p* < 0.001, Figure 2C), to discriminate different stages of breast cancer patients from the controls (stage I: AUC = 0.672, *p* = 0.022; stage II: AUC = 0.874, *p* < 0.001; stage III: AUC = 0.793, *p* = 0.002). In addition, the clinicopathological parameters also demonstrated that the expression of exosomal miR-92b-5p was significantly correlated with stage (*p* = 0.033), estrogen receptor (ER) status (*p* = 0.047), progesterone receptor (PR) status (*p* = 0.007), and epidermal growth factor receptor 2 (HER-2) status (*p* = 0.038) (Table 2), indicating that exosomal miR-92b-5p could be considered a non-invasive body fluid biomarker for breast cancer detection.

### 2.3. miR-92b-5p Was Upregulated in Both Cell Lines and Exosomes of 231-CD63

**Figure 3:**
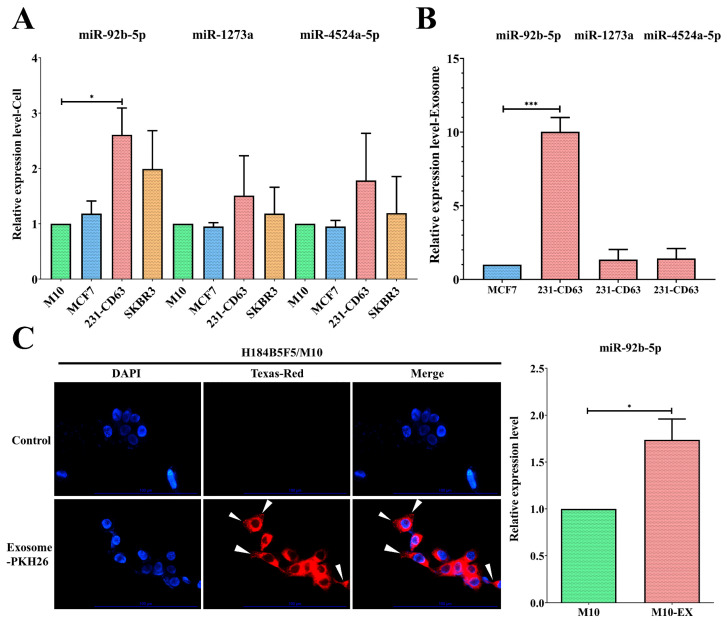
miR-92b-5p exhibits upregulation in both breast cancer cell lines and exosomes. (**A**) Among the three candidate miRNAs, miR-92b-5p is significantly upregulated in the 231-CD63 cell line compared to the control M10 cell line. (**B**) Compared to the exosomes isolated from benign MCF-7, only miR-92b-5p exhibits an increase in the exosomes isolated from 231-CD63. (**C**) Exosomes isolated from 231-CD63 are labeled with PKH-26 and added to the medium of H184B5F5/M10 (M10) normal human mammary epithelial cells (Scale bar: 100 μm). The uptake of exosomes after 6 h is indicated by an arrow mark and is concomitant with the increased miR-92b-5p after 48 h of exosome uptake (M10-EX). Experiments using real-time PCR have been performed in triplicate. The *p*-value is estimated using a two-tailed *t*-test, and a *p*-value of less than 0.05 (denoted as * *p* < 0.05, and *** *p* < 0.001) denotes statistical significance.

In addition to clinical serum validation, we utilized a cell line model to analyze these three exosomal miRNAs in vitro. Compared to the normal M10 cell, only miR-92b-5p was significantly increased in 231-CD63 cells (Figure 3A). We further analyzed exosome levels by comparing exosomes isolated from MCF7 and 231-CD63, and only exosomal miR-92b-5p was overexpressed in the exosomes, indicating that miR-92b-5p was the crucial miRNA that was upregulated in both the breast cancer cell lines and the exosomes (Figure 3B). As miR-92b-5p was upregulated in the clinical breast cancer patients’ serum, the in vitro cell lines, and the exosomes, we focused on it in the next experiment to validate the potential target of exosomal miR-92b-5p. We selected the normal M10 cell as our recipient cell and delivered the PKH-26-labeled exosome. As shown in Figure 3C, the exosomes were taken up by the recipient M10 cell, as shown by the PKH-26 signal; concomitantly, miR-92b-5p was upregulated when the exosomes were taken up by the recipient M10 cell (M10-EX).

### 2.4. MTSS1L Was Targeted by miR-92b-5p

**Figure 4:**
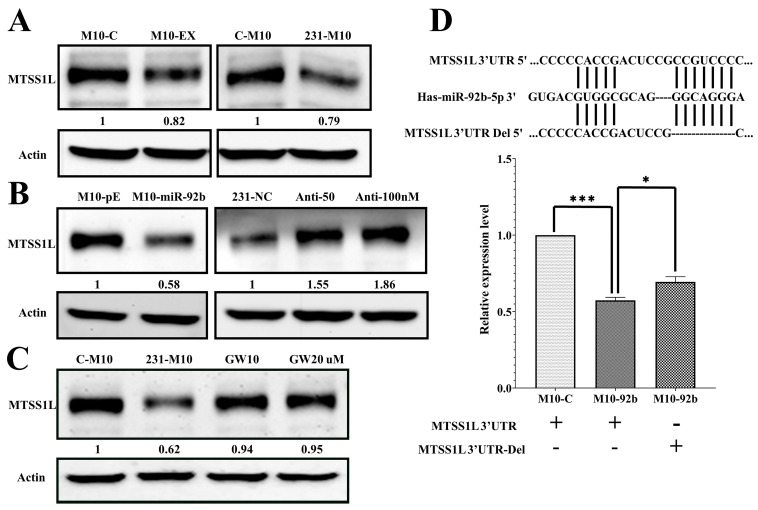
MTSS1L is the targeted gene of miR-92b-5p. (**A**) The protein level of MTSS1L is reduced after 72 h of exosome uptake (M10-EX) and co-culture with 231-CD63 (231-M10). (**B**) The protein level of MTSS1L is reduced after overexpression of miR-92b-5p (M10-miR-92b); this phenotype is reversed when miR-92b-5p is inhibited with different doses of anti-miR-92b-5p (Anti-50/100 nM). (**C**) Reversal of reduced MTSS1L with co-culture can occur when inhibiting the secretion of exosomes with different doses of GW4869 (GW10/20 µM). (**D**) The miR-92b-5p targeting sequence in the MTSS1L 3′UTR is identified, and a deletion is performed to change the sequence (MTSS1L 3′UTR-Del, upper panel). MTSS1L 3′UTR reporter activity decreased in miR-92b-5p-treated cells compared to the control cells, and deletion of the two miR-92b-5p binding sites reversed miR-92b-5p-repressed reporter activity. Experiments have been performed in triplicate. The *p*-value is estimated using a two-tailed *t*-test, and a *p*-value of less than 0.05 (denoted as * *p* < 0.05, and *** *p* < 0.001) denotes statistical significance.

Next, we investigated the potential target of exosomal miR-92b-5p. To identify the target gene affected by miR-92b-5p, we utilized the miRWalk database and found that MTSS1L was targeted by miR-92b-5p. We verified whether MTSS1L could be affected by miR-92b-5p in exosomes using a different approach. We found that (1) MTSS1L was repressed when the M10 cells were delivered with exosomes or co-cultured with 231-CD63 to mimic the tumor microenvironment (Figure 4A); (2) MTSS1L was repressed and increased when miR-92b-5p was overexpressed or knocked down in the M10 and 231-CD63 cells (Figure 4B).

To further confirm that the expression of MTSS1L in the recipient cells was affected by the secretion of exosomes, the co-culture system of the upper transwell insert was treated with a different dose of GW4869, which inhibited the secretion of exosomes [16]. As shown in Figure 4C, MTSS1L was significantly reversed when inhibiting the secretion of exosomes. Indeed, it proved that the downregulation of MTSS1L in the co-culture system was due to the secretion of exosomes from 231-CD63. We further verified that miR-92b-5p could target MTSS1L directly by using the MTSS1L 3′UTR reporter assay. As shown in Figure 4D, the MTSS1L 3′UTR reporter activity of the miR-92b-5p-treated cells was decreased compared to the control cells, and the deletion of the two miR-92b-5p binding sites reversed the miR-92b-5p-repressed reporter activity.

### 2.5. Exosomes Could Alter the Characteristics of the Recipient Cell

**Figure 5:**
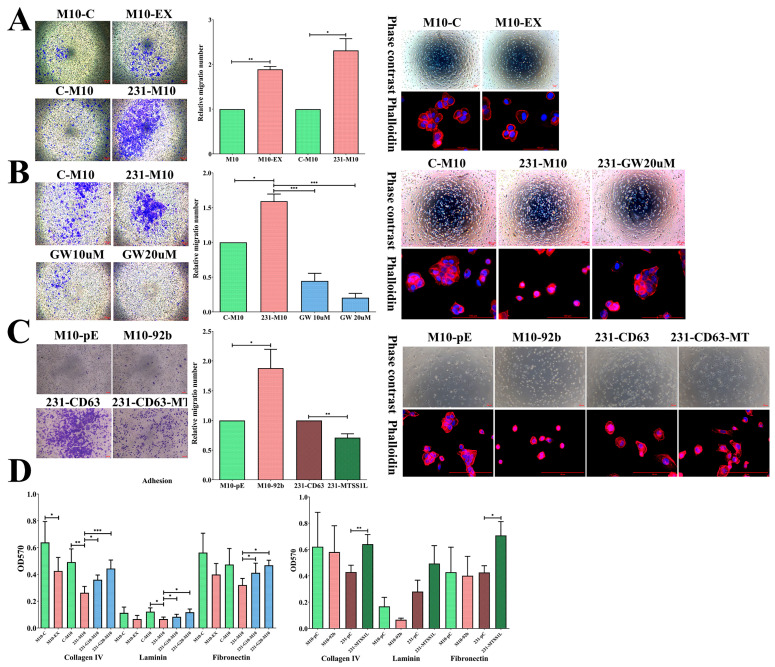
Exosomes alter the migratory, spreading, and adhesion abilities of recipient cells, potentially attributed to MTSS1L in exosomal miRNA. (**A**) The migration ability of recipient M10 cells is increased with exosome uptake (M10-EX) or co-culture with 231-CD63 (231-M10); this is concomitant with an alternation in spreading ability. (**B**) The increased migration ability of recipient M10 cells with co-culture can be significantly repressed when exosome secretion is blocked with GW4869; this is concomitant with a reversed spreading ability. (**C**) Overexpression of miR-92b-5p increases migration and alters the spreading ability of recipient M10 cells (M10-92b). This effect is repressed when MTSS1L is overexpressed in 231-CD63 (231-CD63-MT) and inhibits migration and spreading abilities. Scale bar: 100 μm. (**D**) The adhesion ability is reduced during incubation with exosomes or co-culture with 231-CD63; this can be reversed by blocking exosome secretion. This effect is also observed in the overexpression of miR-92b-5p and reversed with overexpression of MTSS1L in 231-CD63. Experiments have been performed in triplicate. A *p*-value of less than 0.05 (denoted as * *p* < 0.05, ** *p* < 0.01, and *** *p* < 0.001) was calculated by comparing each group to the control, including M10-C, C-M10, M10-pC, and 231-pC.

Exosomes are secreted from the donor cell and taken up by the recipient cell to alter some characteristics of the recipient cell. We found that the recipient normal M10 cells could significantly increase their migration ability when delivered with exosomes (M10-EX) or co-cultured with 231-CD63 (231-M10, Figure 5A). To further confirm that the alteration of the migration ability was due to the secretion of exosomes, different doses of GW4869 were used in co-culture with 231-CD63. As shown in Figure 5B, the migration ability of recipient normal M10 cells was significantly diminished when the secretion was blocked, indicating that the induced effect was due to the secretion of exosomes from 231-CD63.

We also observed the morphology of the recipient cells. Cell spreading is a short-term effect in which transformed cells display reduced adhesion ability and small, round cells with a filopodia structure that fail to spread extensively compared to normal cells [17]. We found that the morphology of M10 was rounder and there were more filopodia structures at the cell edge (M10-EX, 231-M10); however, in the control and GW4869 treatment groups, the morphology tended to be flatter and had more lamellipodia structures (Figure 5A,B, right), indicating the transformation of the recipient normal M10 cells. Likewise, the adhesion ability of the recipient normal M10 cells was also reduced in both those delivered with exosomes and those co-cultured with 231-CD63. This effect was counteracted after GW4869 treatment (Figure 5D, left).

To further realize the functional alternation of MTSS1L and exosomal miRNA, we established two stable cell lines with M10-92b (overexpression of miR-92b-5p in normal M10 cells to mimic the increased expression of miR-92b-5p due to the uptake of exosomes) and 231-CD63-MT (overexpression of MTSS1L in 231-CD63). Overexpression of miR-92b-5p also increased the migration ability and altered the morphology of spreading. In contrast, the migration ability was reduced and the morphology was flatter in 231-CD63-MT (Figure 5C). Likewise, the adhesion ability was reduced in M10-92b-5p cells and counteracted in 231-MTSS1L cells (Figure 5D, right). Together, these results indicated that exosomes could alter the migration, spreading, and adhesion abilities of normal M10 cells, in which these effects may result from MTSS1L downregulation by exosomal miR-92b-5p.

### 2.6. MTSS1L Expression Correlated with the Survival of Breast Cancer Patients

**Figure 6:**
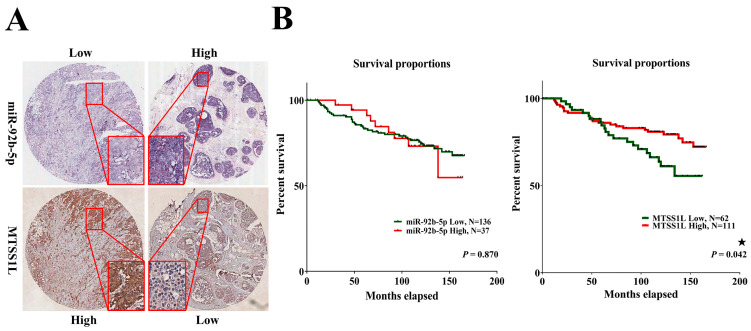
Patients with breast cancer exhibiting high expression of MTSS1L have a favorable 10-year survival rate. (**A**) The representative figure shows that in situ hybridization of miR-92b-5p expression has an inverse correlation with MTSS1L protein level in breast cancer tissues. (**B**) Patients with breast cancer exhibit improved 10-year survival rates when MTSS1L is highly expressed. The survival rates are estimated using the Kaplan–Meier estimator, and the survival difference is tested using the log-rank test. A *p*-value of less than 0.05 (denoted as ★ *p* < 0.05) denotes statistical significance.

In the cell line model, we elucidated MTSS1L as the target of miR-92b-5p, and it was affected by exosomes to impact migration, spreading, and adhesion. Therefore, we further investigated the expression of miR-92b-5p and MTSS1L in clinical breast cancer patient tissues. MiR-92b-5p was analyzed using ISH and MTSS1L using IHC in a total of 187 tissues. MiR-92b-5p was indeed significantly inversely correlated with MTSS1L (*p* = 0.001, Figure 6A and Table 3); however, an interesting phenomenon was that tissue miR-92b-5p had low expression in most of the cases in stage II-III (Table 3). This phenomenon contrasted with serum exosomal miR-92b-5p, which was highly expressed in stage II-III (Table 2).

**Figure 7:**
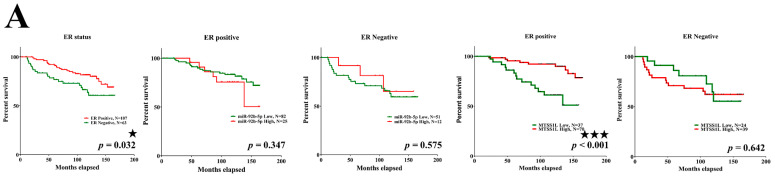

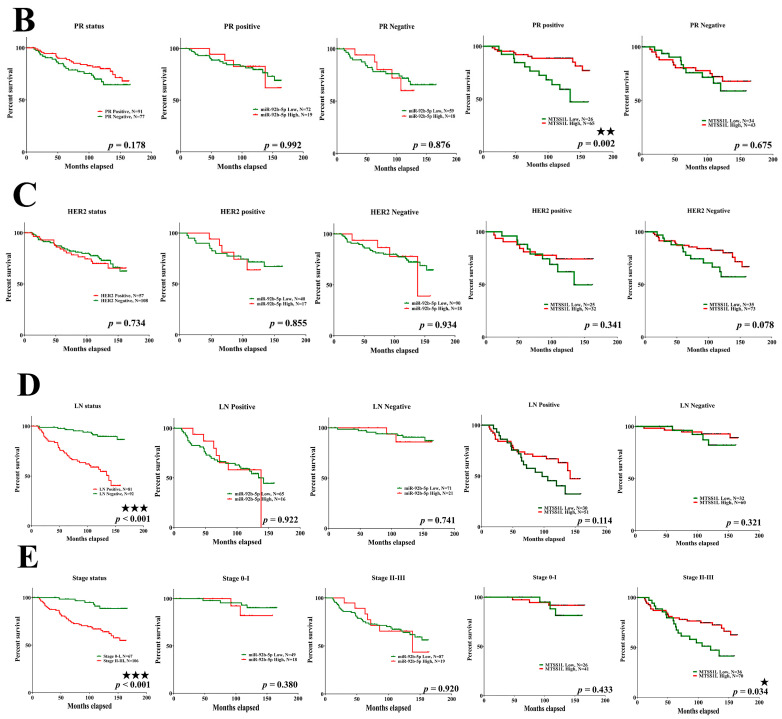
The relationship among miR-92b-5p, MTSS1L, and different receptor statuses. (**A**–**E**) Patients with ER-positive (*p* < 0.001), PR-positive (*p* = 0.002), and stage II–III (*p* = 0.034) breast cancer with high expression of MTSS1L exhibit improved 10-year survival rates. Column 1: all patients with different receptor/LN/stage status; column 2: miR-92b expression level and receptor positive/LN positive/stage 0–I status; column 3: miR-92b expression level and receptor negative/LN negative/stage II–III status; column 4: MTSS1L expression level and receptor positive/LN positive/stage 0–I status; column 5: MTSS1L expression level and receptor negative/LN negative/stage II–III status. The survival rates are estimated using the Kaplan–Meier estimator, and the survival difference is tested using the log-rank test. A *p*-value of less than 0.05 (denoted as ★ *p* < 0.05, ★★ *p* < 0.01, and ★★★ *p* < 0.001) denotes statistical significance.

**Figure 8:**
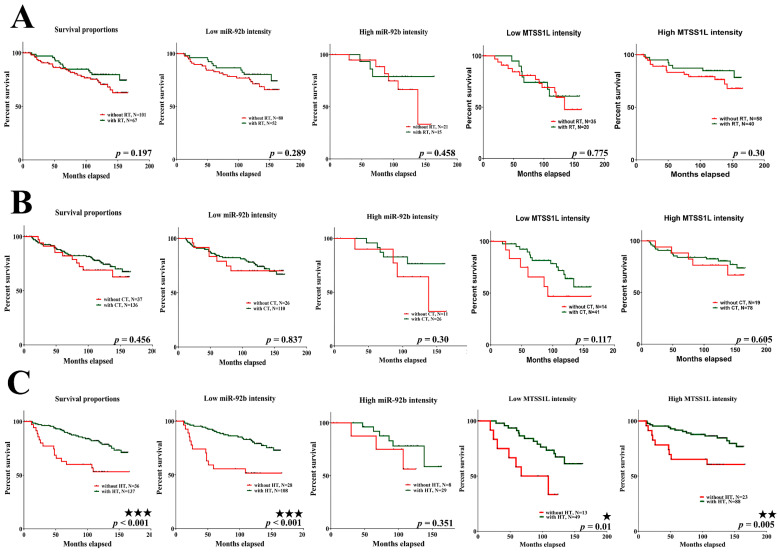
The relationship among miR-92b-5p, MTSS1L, and different therapy regimens. (**A**,**B**) The expression of miR-92b-5p and MTSS1L is not correlated with the survival of RT/CT in breast cancer patients. (**C**) Patients with breast cancer undergoing hormone therapy (HT) with low expression of miR-92b-5p (*p* < 0.001) and high MTSS1L expression (*p* = 0.005) exhibit improved 10-year survival rates. Column 1: all patients with different therapy regimens; column 2: low miR-92b expression level with different therapy regimens; column 3: high miR-92b expression level with different therapy regimens; column 4: low MTSS1L expression level with different therapy regimens; column 5: high MTSS1L expression level with different therapy regimens. The survival rates are estimated using the Kaplan–Meier estimator, and the survival difference is tested using the log-rank test. A *p*-value of less than 0.05 (denoted as ★ *p* < 0.05, ★★ *p* < 0.01, and ★★★ *p* < 0.001) denotes statistical significance.

Next, we analyzed 10-year survival rates using different clinicopathological parameters. In total, 187 cases had 173 cases with recorded survival rates. We found that the expression of MTSS1L was significantly correlated with 10-year survival rates and that high expression of MTSS1L was associated with better survival rates (*p* = 0.042, Figure 6B). We further analyzed the relationship among miR-92b-5p, MTSS1L, different receptor statuses, and therapy regimens. The expression of MTSS1L was significantly correlated with ER-positive status (Figure 7A, *p* < 0.001), PR-positive status (Figure 7B, *p* = 0.002), and stage II-III (Figure 7E, *p* = 0.034), indicating that highly expressed MTSS1L had better survival rates. In the therapy regimen, low expression of miR-92b-5p with hormone therapy (HT) had better survival than that without HT (*p* < 0.001), and high expression of MTSS1L with HT had better survival (*p* = 0.005) than low expression of MTSS1L with HT (*p* = 0.01), indicating that MTSS1L may be involved in the survival of breast cancer patients on hormone therapy (Figure 8C).

## 3. Discussion

Studies regarding exosomal miRNAs as non-invasive body fluid biomarkers for cancer detection have been growing in recent years [18,19,20]. The abundance of exosomes is enriched in cancer patients compared to healthy controls [21,22], making exosomal miRNA an ideal, novel, non-invasive biomarker for cancer detection. Until now, there have been few studies investigating exosomal miRNAs as diagnostic markers for breast cancer [20]. The combination of exosomal miR-1246 and miR-21 could differentiate patients with breast cancer from healthy subjects with an AUC of 0.73 [23]. An exosomal miR-106a-363 cluster was another diagnostic biomarker in which four plasma miRNAs and four serum miRNAs were constructed to discriminate patients with breast cancer from controls with an AUC > 0.8 [24]. Eichelser et al. demonstrated that exosomal miR-101 and miR-372 were overexpressed in patients with breast cancer compared to healthy controls and that exosomal miR-373 was a unique biomarker for the diagnosis of triple-negative breast cancer [11]. In our study, we found that a single crucial exosomal, miR-92b-5p, could discriminate patients with breast cancer from controls with an AUC of 0.787 and was significantly correlated with stage.

Several studies have demonstrated that miR-92b is a unique oncomir in breast cancer. In tissue sections, miR-92b is a luminal A subtype-specific miRNA that is upregulated in breast cancer [25] and presents a distinct miRNA signature in young women with breast cancer [26]. However, only a few studies have investigated the role of exosomal miR-92b in breast cancer. Exosomal miR-92b-5p had previously been identified as a diagnostic biomarker for heart-related diseases [27,28,29]. In 2013, a plasma miRNA signature was generated through comparison with a previous public dataset, revealing that miR-92b-5p is a unique oncomir in breast cancer compared to lung or colorectal cancer [30]. The above study is the only one to investigate the role of miR-92b-5p in cancer and indicates that circulating miR-92b-5p may play a crucial role in breast cancer. To the best of our knowledge, we have provided the first evidence that exosomal miR-92b-5p may function as a diagnostic biomarker and discriminate different stages of breast cancer patients from controls. We have also elucidated its function by identifying its unique target, MTSS1L.

In spite of the diagnostic values of exosomal miRNA, in most studies, the function of these exosomal miRNAs is not fully understood. In our study, we found that a unique target of MTSS1L was affected by miR-92b-5p to impact the migration, spreading, and adhesion of recipient normal human mammary epithelial cells and survival rates in clinical tissues. MTSS1L is a rarely studied member of the inverse BAR (Bin, Amphiphysin, and Rvs, I-BAR) superfamily, which contains MIM-like members (MTSS1L, MIM) and IRSp53 members (IRSp53, IRTKS, and FLJ22582). MTSS1L has two major functional domains for a scaffolding function—including the WASP-homology 2 (WH2) domain of the actin-binding motif and the IRSp53-MIM domain (IMD), which is also called the I-BAR domain—to promote the formation of dynamic membrane tubules or membrane protrusions of lamellipodia and filopodia [31,32]. MTSS1L is an actin dynamics regulator that promotes lamellipodia formation and cell spreading [33,34]. Recently, another study has shown that MTSS1L may be involved in membrane curvature, dendritic spine formation, and synaptic function [35]. However, to the best of our knowledge, there are no studies concerning the role of MTSS1L in cancer. In this study, we provide the first evidence of the role of MTSS1L in cancer by using in vitro cell lines and clinical tissues.

In the in vitro cell lines (as part of the role of the actin dynamics regulator MTSS1L), the migration, adhesion, and spreading ability of recipient normal M10 cells were impacted by exosome uptake, co-culture with 231-CD63 cells, and miR-92b-5p, all concomitant with the inhibition of MTSS1L. Thus, the transformation ability of exosomes toward recipient normal M10 cells may be involved in the axis of miR-92b-5p to MTSS1L. In the clinical tissues, high expression of MTSS1L has better 10-year survival rates. Metastasis is a crucial process that reduces survival rates and leads to death in most cancer patients. In the initiation of metastasis, cell migration is the first step that dysregulates and promotes the dissemination of tumor cells from the primary site to secondary sites. This dysregulation of migration depends on the dynamics of cytoskeletal proteins such as actin and the formation of different protrusions such as filopodia that promote migration, leading to metastasis and low survival rates [36]. We also found under hormone therapy that patients with high expression of MTSS1L have better survival rates than those with low expression of MTSS1L. One interesting study demonstrated that hormone therapy might increase local invasion of the tumor by decreasing cytoskeletal-related protein EVL and that estrogen may prevent metastasis through actin cytoskeletal remodeling [37], indicating that the expression of cytoskeletal-related proteins is crucial in controlling metastasis under hormone therapy. Taken together, the actin dynamics regulator MTSS1L affects cell migration, adhesion, and spreading in vitro and prolongs survival in clinical patients, which may be due to the involvement of metastasis control and cytoskeletal remodeling, indicating that MTSS1L may function as a metastasis suppressor in cancer.

In addition to circulating exosomal miR-92b-5p from blood, we also analyzed miR-92b-5p expression in clinical tissues using ISH and found that miR-92b-5p was indeed significantly inversely correlated with MTSS1L; however, an interesting phenomenon was that the expression of miR-92b-5p was reduced in most of the cases in stage II-III, which differed from the serum of exosomal miR-92b-5p. This opposite expression of exosomal miRNA and tissue miRNA has been observed in several studies, indicating that exosomal miRNA does not mirror tissue miRNA. One study found a different expression pattern in exosomal miRNAs and tissue miRNAs in breast cancer patients with and without recurrence [38]. Another study provided a comprehensive analysis of exosomal/plasma/tissue miRNAs in tongue cancer, and different tissue miRNAs were observed in exosomes or plasma [39]. Until now, the mechanisms controlling the secretion of miRNAs into exosomes or their retention in tissues remain unknown; however, tumors themselves may release miRNAs into circulation [9]. In addition, exosomal miRNAs can reflect a more comprehensive response to cancer and are more stable and reliable in diagnosis [10,11,38,39].

## 4. Materials and Methods

### 4.1. Cell Lines and Reagents

The human breast cancer cell lines MDA-MB-231, MCF-7, and SKBR3, as well as H184B5F5/M10 (M10) normal human mammary epithelial cells were purchased from the Bioresource Collection and Research Center (Hsinchu, Taiwan). Immortalized human breast fibroblasts (RMF-EGs) were kindly provided by Dr. Charlotte Kuperwasser and Dr. Kelvin Kun-Chih Tsai. These cells were cultured in DMEM/F12 containing 10% fetal bovine serum (FBS). MDA-MB-231-CD63-RFP (231-CD63) was established by transfection with pCT-CD63-RFP constructs (System Biosciences, Mountain View, CA, USA) and selected with puromycin. MiR-92b-5p and MTSS1L expression vectors were purchased from GeneCopoeia (Rockville, MD, USA). Anti-miR-92b-5p, negative control, and siPORT NeoFX were obtained from Thermo Fisher Scientific (Waltham, MA, USA). GW4869 was purchased from Cayman (Ann Arbor, MI, USA).

### 4.2. Study Population

A total of 112 female participants were recruited into this study for serum collection. This included 53 control volunteers and 59 breast cancer patients. Of the 59 patients, 21 had stage I, 27 had stage II, and 11 had stage III breast cancer. All serum samples were collected before receiving any hormone, chemotherapy, or radiotherapy and obtained from the Division of Breast Oncology and Surgery, Kaohsiung Medical University Hospital, with approval from the Internal Review Board and informed consent from all patients (KMUH-IRB-990174, KMUHIRB-F(I)-20200107).

### 4.3. Exosome Isolation and Labeling

231-CD63 was cultured in a 10 cm dish with a high density of 2  ×  10^6^ cells for 24 h and then replaced with a serum-free medium for 48 h. The culture medium was then centrifuged at 4000 rpm for 10 min, and exosomes were isolated using ExoQuick-TC Exosome Precipitation Solution (System Biosciences, Mountain View, CA, USA) according to the manufacturer’s protocol. Exosome labeling was carried out by labeling with PKH26 dye (Sigma, St. Louis, MO, USA) according to the manufacturer’s protocol. Exosomes isolated from serum were centrifuged at 13,000 rpm for 10 min and precipitated with ExoQuick Exosome Precipitation Solution (System Biosciences, Mountain View, CA, USA) according to the manufacturer’s protocol.

### 4.4. Microarray Analysis

RNA was extracted using TRIzol reagent, and microarray analysis was conducted using Phalanx Human miRNA OneArray (Phalanx Biotech Group, Hsinchu, Taiwan). The differentially expressed genes were identified following the criteria of log2 fold change ≥0.8 and *p* < 0.05.

### 4.5. Western Blots

Cells and exosomes were lysed in RIPA buffer with an appropriate protease inhibitor at 4 °C for 20 min. Protein concentrations were normalized using BCA protein assay, boiled in sample buffer, separated using SDS-polyacrylamide gel electrophoresis (SDS-PAGE), and then transferred to nitrocellulose paper. Membranes were blocked with non-fat dry milk in Tris-buffered saline containing 0.05% Tween 20 (TBST) and then incubated with primary antibodies against CD63 (System Biosciences, Mountain View, CA, USA), RFP (SignalChem, Richmond, CA, USA), Hsp70, Calnexin (Genetex, Hsinchu, Taiwan), MTSS1L (Bethyl Laboratories, Montgomery, TX, USA), and Actin (Millipore, Billerica, MA, USA). Enhanced chemiluminescence reagents (PerkinElmer, Shelton, CT, USA) were used to depict the protein bands on the blots and visualized using UVP biospectrum 600. Western blot quantification was performed using the Image J software version 1.52 (National Institutes of Health, MD, USA).

### 4.6. Exosome RNA Extraction and Real-Time PCR

Total RNA was isolated from cells using TRIzol reagent, and exosome RNA was extracted using SeraMir Exosome RNA Amplification kit (System Biosciences, Mountain View, CA, USA) according to the manufacturer’s protocol. cDNA was synthesized using the Mir-X miRNA First-Strand Synthesis Kit (TaKaRa, Kusatsu, Shiga, Japan) for microRNA quantification. The target miRNA sequence was synthesized according to miRBase and quantified using real-time PCR with SYBR green GoTaq qPCR Master Mix (Promega, Madison, WI, USA), and U6 small nuclear RNA was used as an internal control for microRNA quantification.

### 4.7. Nanoparticle Tracking Analysis (NTA)

The extract size distribution and concentration of exosomes were analyzed using ZetaView PMX 120 (Particle Metrix, Meerbusch, Germany) on the basis of light scattering and Brownian motion, and the results were analyzed using NTA software version 3.2.

### 4.8. Transmission Electron Microscopy (TEM)

A purified exosome was dried onto freshly glow-discharged formvar/carbon grids and negatively stained with 1% uranyl acetate. The samples were visualized using TEM with an FEI Tecnai G2 F20 S-TWIN.

### 4.9. Co-Culture System

Recipient cells of M10 were placed in the lower 6 wells and 231-CD63 cells in the upper wells of a 1 μm polyester membrane transwell insert (Corning, Manassas, VA, USA). This allowed the 231-CD63 cells to only be in contact with the culture medium of the recipient cells.

### 4.10. 3′-UTR Reporter and Dual-Luciferase Assay

Cells were transfected with the 3′-UTR reporters of MTSS1L (Genecopoeia, Rockville, MD, USA). Deletions were introduced into the two predicted CCGTCCC binding sites of miR-92b-5p, which were manufactured by Genecopoeia, to delete the CCGTCCC targeting sequence. After 48 h, the medium was removed, and the cells were washed with cold PBS and dissolved in Passive Lysis Buffer (Promega, Madison, WI, USA), and 20 μL of cell lysate was transferred into a luminometer plate. Luciferase activity and Renilla luciferase activity (as an internal control) were measured using dual-luciferase reporter assay (Promega, Madison, WI, USA) and TECAN Infinite 200 pro. MTSS1L 3′-UTR reporter activity was normalized by Renilla luciferase activity and protein concentrations, and the results from three independent assays in different cell lines were compared.

### 4.11. Migration and Spreading Assay

An in vitro migration assay was performed using 24-well transwell units with polycarbonate filters coated on the upper side, and 2 × 10^4^ cells were calculated in 100 μL of serum-free medium and placed onto the upper part of the transwell unit. After 24 h, non-migrated cells on the upper part of the membrane were removed with a cotton swab. Cells that migrated to the bottom surface of the membrane were fixed with formaldehyde, stained with Giemsa solution, and counted under a microscope. In the spreading assay, 1 × 10^5^ cells were calculated and placed onto the fibronectin-coated coverslips in 24 wells and cultured at 37 °C for 6 h, washed with PBS, fixed with 4% paraformaldehyde in PBS for 10 min, permeabilized with 1% Triton X-100 for 10 min, and blocked with 5% bovine serum albumin in PBS for 30 min. The cells were then incubated with Alexa Fluor 594 Phalloidin (Invitrogen, Carlsbad, CA, USA) for 30 min according to the manufacturer’s protocol and imaged with a Leica DM IL LED and Leica DMI 4000 fluorescent microscope (Hesse, Wetzlar, Germany).

### 4.12. Cell Adhesion Assay

Collagen IV, fibronectin, and laminin (Sigma, St. Louis, MO, USA; 5 μg/100 μL) were coated onto 96-well ELISA plates at 4 °C overnight. Cells at a density of 20,000/well were added to each well and incubated at 37 °C for 90 min. Wells were washed with PBS, fixed with 4% formaldehyde, and air-dried. Adhesive cells were stained with crystal violet (0.05%) and de-stained with DMSO. The optical density of each well was measured using an ELISA reader at 570 nm and normalized with a blank well (without extracellular matrix).

### 4.13. In Situ Hybridization (ISH) and Immunohistochemical Staining (IHC)

Human breast cancer tissue was obtained from patients who had undergone surgical operations at the Division of Breast Oncology and Surgery, Kaohsiung Medical University Hospital, with de-linkage FFPE samples. All samples received approval from the Internal Review Board, and informed consent from all patients was obtained (KMUH-IRB-990174, KMUHIRB-F(I)-20200107). The procedure was performed according to the manufacturer’s protocol for the IsHyb in situ hybridization kit (BioChain, Newark, CA, USA). Briefly, the slides were treated with 10 μg/mL Proteinase-K at 37 °C for 10 min. The miR-92b-5p LNA probe (Exiqon, Germantown, MD, USA) was denatured at 90 °C for 5 min, diluted to 50 nM, and incubated with the slides at 55 °C for 120 min. The slides were incubated with 1:200 AP-conjugated anti-DIG antibody at 4 °C overnight, and the substrate NBT-BCIP was added and incubated in the dark for 1 h to develop a blue signal. The nuclei were counterstained with Fast Red. The intensity of staining was graded as 0: negative; 1: mild; 2: moderate; and 3: strong.

In immunohistochemical staining, the procedure was performed according to the manufacturer’s protocol for Mouse/Rabbit PolyDetector HRP with the DAB kit (BioSB, Santa Barbara, CA, USA). Briefly, the slides were blocked for 10 min with 3% hydrogen peroxide to deprive endogenous peroxidase activity, and antigen retrieval was conducted by incubating the slides in 0.01 M citrate buffer (pH 6) with a microwave. The tissues were incubated with anti-MTSS1L antibody (Bethyl Laboratories, Montgomery, TX, USA, 1:100), detected with horseradish peroxidase-conjugated secondary antibody, and then developed using diaminobenzidine substrate (DAB). The intensity of staining was graded as 0: negative; 1: mild; 2: moderate; and 3: strong.

### 4.14. Statistical Analysis

Appropriate statistical analyses were performed using Graphpad Prism 9 (Graphpad Software, Inc., La Jolla, CA, USA). For statistical analyses, a comparison of the different groups was performed using a two-tailed *t*-test and a chi-squared test. A *p*-value less than 0.05 (denoted as * *p* < 0.05, ** *p* < 0.01, and *** *p* < 0.001) was considered statistically significant. The specificity and sensitivity of the markers were analyzed using the receiver operating characteristic curve (ROC curve), and the area under the curve (AUC) was calculated.

## 5. Conclusions

In conclusion, this study has demonstrated that exosomal miR-92b-5p has clinical potential as a non-invasive body fluid biomarker for breast cancer detection. MiR-92b-5p affects the migration, adhesion, and spreading ability of recipient cells through the downregulation of MTSS1L. Additionally, we provide the first evidence of the role of MTSS1L in cancer. High MTSS1L expression is associated with better 10-year overall survival rates in patients undergoing hormone therapy. The expression of MTSS1L was significantly inversely correlated with tissue miR-92b-5p. These results indicate the clinical potential of exosomal miR-92b-5p and novel therapeutic strategies in the axis of miR-92b-5p to MTSS1L for the control of metastasis and improvement in patient survival.

## Figures and Tables

**Table 1:** Total 88 serum exosomal miRNAs from microarray data and the literature detected by real-time PCR.

Ct < 35	Ct ≥ 35			
miRNA ID	miRNA ID	miRNA ID	miRNA ID	miRNA ID
miR-21	miR-7	miR-140	miR-204-5p	miR-502-5p
miR-92a-3p	miR-10b	miR-141	miR-204-3p	miR-503
miR-92b-5p	miR-17	miR-142-3p	miR-205	miR-503-3p
miR-223-3p	miR-18a	miR-142-5p	miR-214	miR-548d-3p
miR-320a	miR-19a-3p	miR-146a	miR-221	miR-550a-5p
miR-483-3p	miR-20a	miR-148a	miR-222	miR-602
miR-1273a	miR-22-3p	miR-148b	miR-223-5p	miR-638
miR-4524a-5p	miR-23a	miR-149	miR-302a	miR-642a-3p
miR-4634	miR-34a	miR-149-3p	miR-302b	miR-654-5p
	miR-34b	miR-150	miR-326	miR-744-5p
	miR-92a-1-5p	miR-181a	miR-361	miR-765
	miR-96	miR-181b	miR-371a-5p	miR-2278
	miR-101-3p	miR-182	miR-371a-3p	miR-3145-5p
	miR-101-5p	miR-183	miR-372-5p	miR-3146
	miR-105	miR-186	miR-372-3p	miR-3149
	miR-122-5p	miR-197-3p	miR-373-5p	miR-3679-5p
	miR-125a	miR-199b	miR-373-3p	miR-4644
	miR-128b	miR-200a	miR-449b-3p	miR-4653-3p
	miR-133a	miR-202-3p	miR-483-5p	miR-4725-3p
	miR-133b	miR-203	miR-484	

**Table 2:** Correlation of exosomal miR-92b-5p and clinicopathological parameters of breast cancer.

		Exosomal miR-92b-5p			*p*-Value
		Total	≤2	>2	
Stage	0–I	21	10	11	0.033 *
	II–III	38	8	30	
Lymph node metastasis	Positive	24	5	19	0.181
	Negative	35	13	22	
ER	Positive	43	10	33	0.047 *
	Negative	16	8	8	
PR	Positive	35	6	29	0.007 **
	Negative	24	12	12	
HER-2	≥3	13	7	6	0.038 *
	<3	46	11	35	
Subtypes	Non-TNBC	52	15	37	0.45
	TNBC	7	3	4		
Subtypes	ER-positive	39	8	31	0.023 *
	HER-2-positive	13	7	6		

* *p* < 0.05, and ** *p* < 0.01.

**Table 3:** Correlation of tissue miR-92b-5p and clinicopathological parameters of breast cancer.

		Tissue miR-92b-5p			*p*-Value
		Total	Low	High	
MTSS1L	Low	68	44	24	0.001 **
	High	119	101	18	
Stage	0–I	70	51	19	0.235
	II–III	117	94	23	
Lymph node ^1^ metastasis	Positive	81	65	16	0.649
	Negative	93	72	21	
ER ^2^	Positive	107	82	25	0.51
	Negative	63	51	12	
PR ^3^	Positive	91	72	19	0.697
	Negative	77	59	18	
HER-2 ^4^	≥3	57	40	17	0.049 *
	<3	108	90	18	
Subtypes	Non-TNBC	130	99	31	0.28
	TNBC	33	28	5	
Subtypes	ER-positive	73	59	14	0.16
	HER-2-positive	57	40	17	

^1^ Thirteen cases without records of lymph node metastasis. ^2^ Seventeen cases without records of ER status. ^3^ Nineteen cases without records of PR status. ^4^ Twenty-two cases without records of HER-2 status. * *p* < 0.05, and ** *p* < 0.01.

## Data Availability

Data are contained within the article and Supplementary Materials.

## Supplementary Materials

The supporting information can be downloaded at https://www.mdpi.com/article/10.3390/ijms25021295/s1.
