# Supplementary material for: Exosomal microRNA-92b Is a Diagnostic Biomarker in Breast Cancer and Targets Survival-Related MTSS1L to Promote Tumorigenesis
Source: Int J Mol Sci. 2024 Jan 20;25(2):1295. doi: 10.3390/ijms25021295 (PMC10816035; doi:10.3390/ijms25021295)

Supplementary Materials:

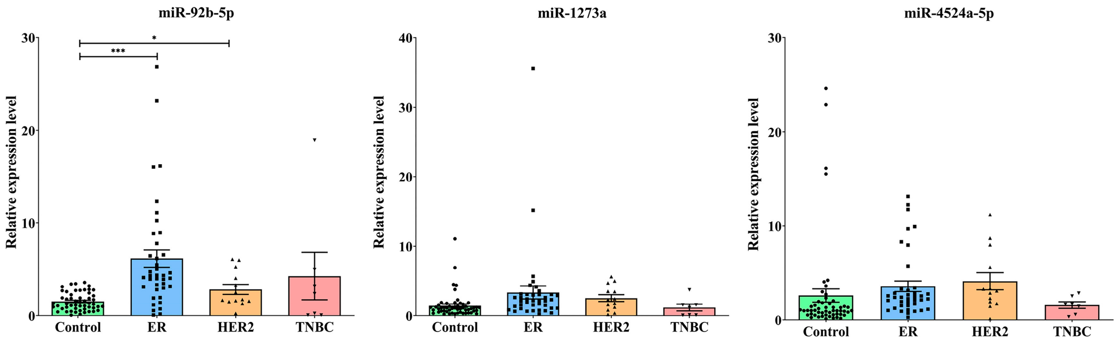

Supplementary Figure S1. Among the three candidate exosomal miRNAs, miR-92b-5p exhibit significant increase in ER and HER2 subtypes patients with breast cancer compared with that of controls. The *p*-value is estimated using a two-tailed t-test, and a *p* value of less than 0.05 (denoted as * $p < 0.05$, and *** $p < 0.001$) denotes statistical significance.

Original Western Blot Figures

Thermo Scientific
PageRuler Prestained Protein Ladder
#26616
Fig 1A

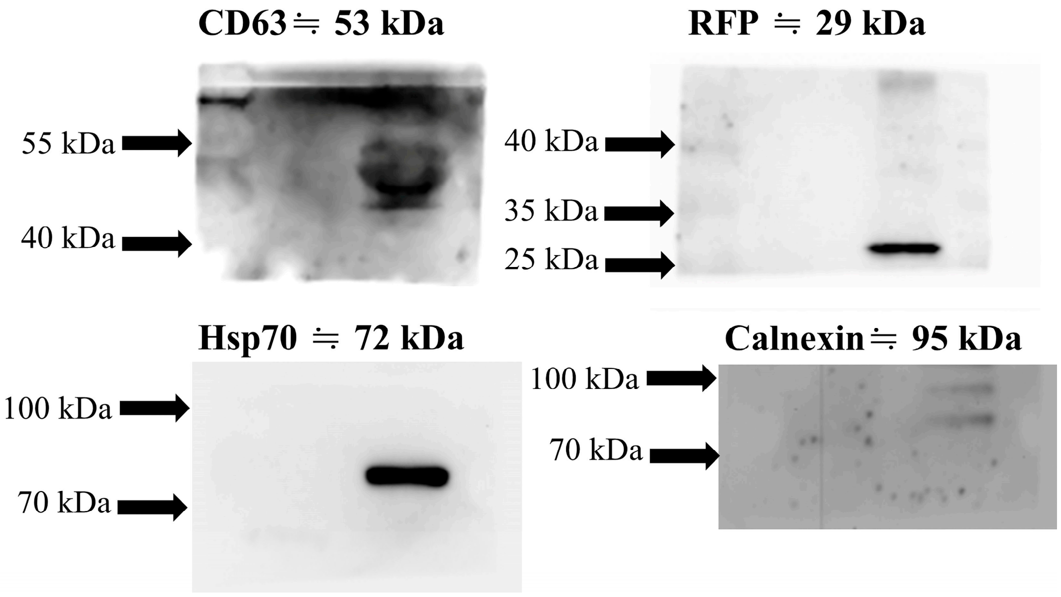

Thermo Scientific
PageRuler Prestained Protein Ladder
#26616

Fig 2A

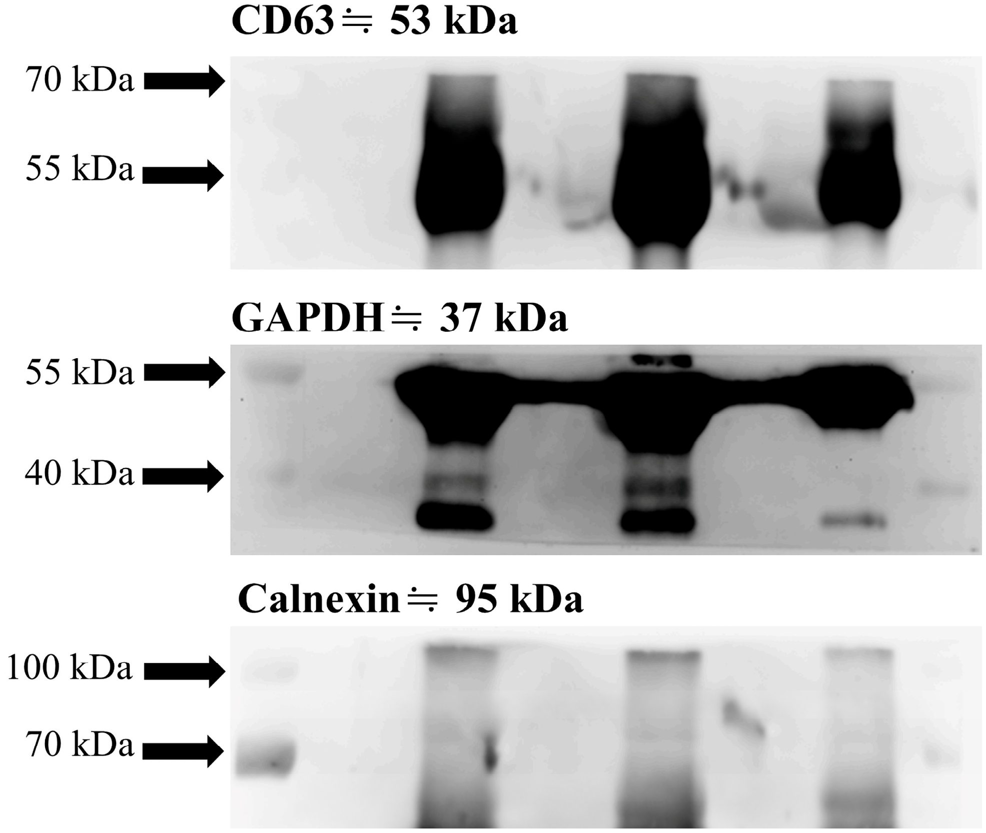

Thermo Scientific PageRuler Prestained Protein Ladder #26616 **Fig 4A-C**

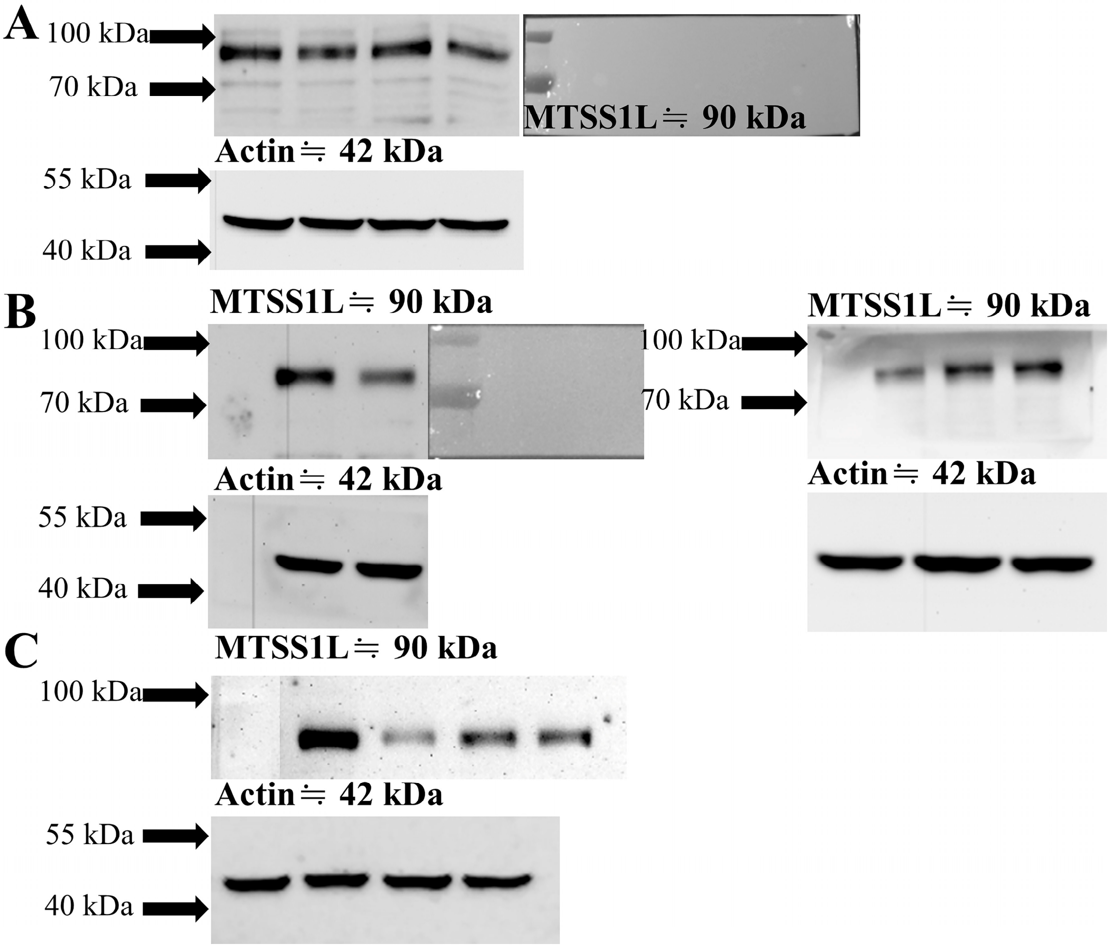

Supplement: Supplementary file 1 [file ijms-25-01295-s001.zip › ijms-2811509-supplementary.pdf]
